# National Arthritis Awareness Month — May 2018

**DOI:** 10.15585/mmwr.mm6717a1

**Published:** 2018-05-04

May is National Arthritis Awareness Month. In the United States, 54 million adults have some form of doctor-diagnosed arthritis (*1*), a number projected to increase to 78 million by 2040.* Approximately two thirds of adults with arthritis have overweight or obesity (*1*), and only 36% meet the recommended aerobic physical activity guidelines.^†^

Engaging in physical activity and maintaining a healthy weight can help manage arthritis symptoms.^§^ Physical activity can reduce arthritis pain, improve function and mood, and delay the onset of disability. Even small amounts of weight loss have been shown to significantly reduce pressure on the joints. Adults who have overweight or obesity and receive weight-loss counseling from a health care provider are approximately four times more likely to attempt to lose weight than are those who do not receive counseling (*2*). Health care providers can play a valuable role by counseling their patients with arthritis to be physically active, lose weight if they have overweight or obesity, and get self-management education (*2*,*3*). A report in this issue found that the percentage of health care providers counseling arthritis patients about weight loss increased significantly from 2002 to 2014 (*3*).

## Footnotes

* https://onlinelibrary.wiley.com/doi/epdf/10.1002/art.39692.

† https://www.sciencedirect.com/science/article/pii/S0749379717302076.

§
https://www.cdc.gov/arthritis/basics/management.htm .
